# Supplementary material for: Content-rich biological network constructed by mining PubMed abstracts
Source: BMC Bioinformatics. 2004 Oct 8;5:147. doi: 10.1186/1471-2105-5-147 (PMC528731; doi:10.1186/1471-2105-5-147)
Supplement: Additional File 2 — The original results of the above study (non-essential files are deleted to keep the file size under the limit set by BMC bioinformatics). [file 1471-2105-5-147-S2.bz2 › chilibotAdditionalFile2/dip05/81ID8798570E293/html/EPHB1_GRB2.html]

 


 **EPHB1** and **GRB2** 
  
Found 15 abstracts in PubMed, retrieved 05.  
 

 What does Google say? 
 PDF only 
| .edu only 

---

**Interactive relationship** (e.g. stimulation, inhibition, etc)

**Neutral relationship**- Both the catalytic activity and, to a much lesser extent, the  **Grb2**  binding tyrosyl phosphorylation sites of SHP 2 are required for maximal FGF 2 induced Erk activity and Elk  [ **EPHB1** ]  1 transactivation.  Ref: 11997521 Mol Cell Biol, 2002
